# Takotsubo syndrome triggered by change in position in a patient with thoracic vertebral fracture: A case report

**DOI:** 10.1097/MD.0000000000024088

**Published:** 2021-01-15

**Authors:** Zhen Zhang, Hao Kong, Si-Yu Zhang, Ting-Ting Guan

**Affiliations:** a Department of Anesthesiology; b Department of Cardiac Surgery, Peking University First Hospital, Beijing, China.

**Keywords:** local anesthesia, percutaneous kyphoplasty, Takotsubo syndrome, thoracic vertebral fracture

## Abstract

**Rationale:**

Takotsubo syndrome (TTS) is characterized by recovery of wall motion abnormalities and acute left ventricular dysfunction, which are often caused by acute physical or emotional stressors. It is rarely reported that TTS can be precipitated by change in position in the patient in the operating room. We report a case of a patient with a thoracic vertebral fracture who presented with TTS precipitated by changing from a supine to a prone position before percutaneous kyphoplasty (PKP) under local anesthesia.

**Patient concerns:**

A 76-year-old man who was diagnosed with a fracture in a thoracic vertebra was sent to the operating room to undergo PKP under local anesthesia. Approximately 5 minutes after changing from a supine to a prone position, which is necessary for PKP, the patient experienced chest pain, headache, and sweating.

**Diagnosis:**

A fracture in a thoracic vertebra; TTS.

**Interventions:**

As a result of 12-lead electrocardiography, echocardiography, left ventriculogram, and cardiac catheterization, the diagnosis of TTS was retained, and supportive therapy was initiated.

**Outcomes:**

Two hours later, the patient's symptoms mitigated significantly and the ST segment returned to baseline. Four days later, echocardiography showed normal systolic function without wall motion abnormalities and the patient returned to the orthopedics ward for further treatment.

**Lessons:**

It is necessary for anesthetists to recognize TTS which is life-threatening during monitored anesthetic care (MAC). We highlight the importance of being alerted to the possibility of TTS when managing patients with thoracic vertebral fractures undergoing surgery under local anesthesia.

## 1 Introduction

Takotsubo syndrome (TTS), which is also known as Takotsubo cardiomyopathy or broken heart syndrome, is reversible cardiomyopathy that was first reported in 1990 in the Japanese population.^[1]^

Accounting for approximately 2% of all cases of suspected acute myocardial infarction (AMI), TTS is characterized by recovery of wall motion abnormalities and acute left ventricular dysfunction, which are often caused by acute physical or emotional stressors that result in excessive catecholamine release.^[1,2]^ Emotional and physical factors may trigger TTS. Emotional triggers include negative events such as bereavement of a close relative, disasters, bullying, and financial loss; and positive events include attending a wedding or birthday party, or winning the lottery.^[2]^ Physical triggers extend from serious diseases (e.g., intracranial hemorrhage, sepsis) to physiological processes (e.g., sexual intercourse, pregnancy).^[3]^ However, it is rarely reported that TTS can be precipitated by a change in position in the patient in the operating room. We report a case of a 76-year-old man with a thoracic vertebral fracture who presented with TTS precipitated by changing from a supine to a prone position before undergoing percutaneous kyphoplasty (PKP) under local anesthesia. Written informed consent was obtained from the patient to present his case and the accompanying images.

## 2 Case report

A 76-year-old man was admitted to our orthopedics ward after being diagnosed with a fracture in a thoracic vertebra. The patient's past medical history included Alzheimer's disease, which was diagnosed 6 years previously; asthma, which was diagnosed 40 years previously; and Budd-Chiari syndrome, which was treated with surgery in 1997.

On admission, the patient's vital signs were as follows: temperature, 36.3°C; pulse, 72 beats per minute; respiratory rate, 20 breaths per minute; and blood pressure, 115/61 mm Hg. A physical examination revealed backache related to thoracic vertebra fracture, which was verified by X-ray. The patient's neurological and cardiopulmonary examinations were considered normal. The results of chest X-ray and electrocardiography (ECG) were also normal. Echocardiography showed a left ventricular ejection fraction of 76.2%. Laboratory test results were as follows: white blood cell count, 5.8 × 10^9^/L; platelet count, 127 × 10^9^/L; hemoglobin, 152 g/L; prothrombin time, 11.9 seconds; activated partial thromboplastin time, 30.9 seconds; international normalized ratio, 1.03; aspartate aminotransferase, 23 IU/L; alanine aminotransferase, 18 IU/L; creatinine, 80.7 μmol/L; blood urea nitrogen, 7.59 mmol/L; natremia, 140 mmol/L; kalemia, 4.38 mmol/L; cardiac troponin, 0.005 ng/mL; and brain natriuretic peptide, 59 pg/mL.

**Figure 1:**
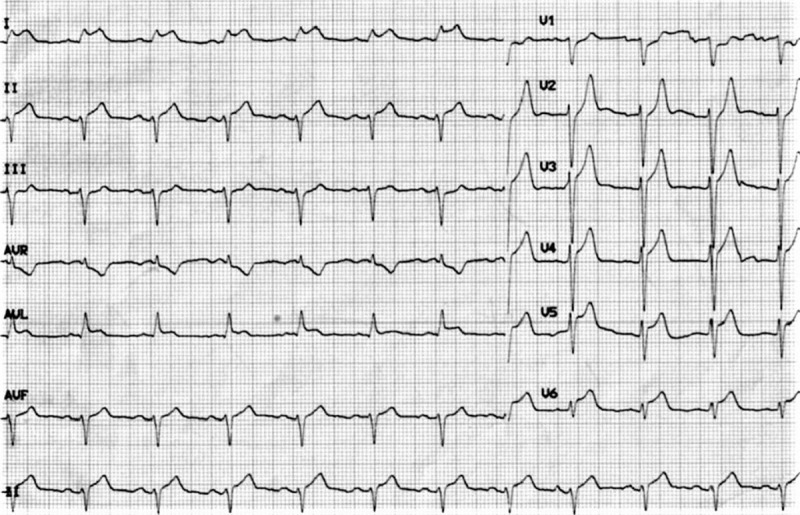
12-Lead electrocardiogram in the operating room shows ST segment elevation at derivations I, avL, V3, V4, V5, and V6.

On day 2, the patient was sent to the operating room to undergo PKP under local anesthesia to treat the thoracic vertebral fracture. Approximately 5 minutes after changing from a supine to a prone position, which is necessary for PKP, the patient experienced chest pain, headache, and sweating. Simultaneously, ST segment elevation was observed on ECG. Considering the possibility of AMI, we examined the patient by 12-lead ECG, which showed ST segment elevation at derivations I, aVL, V3, V4, V5, and V6 (Fig. 1). After administration of loading doses of aspirin (300 mg) and clopidogrel (300 mg), the patient was immediately transferred to the coronary care unit.

**Figure 2:**
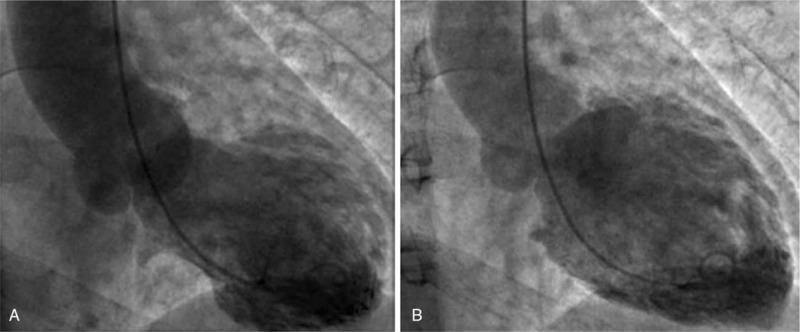
Left ventriculogram during systole (A) and diastole (B). These figures show the characteristic apical ballooning indicative of TTS. TTS, Takotsubo syndrome.

**Figure 3:**
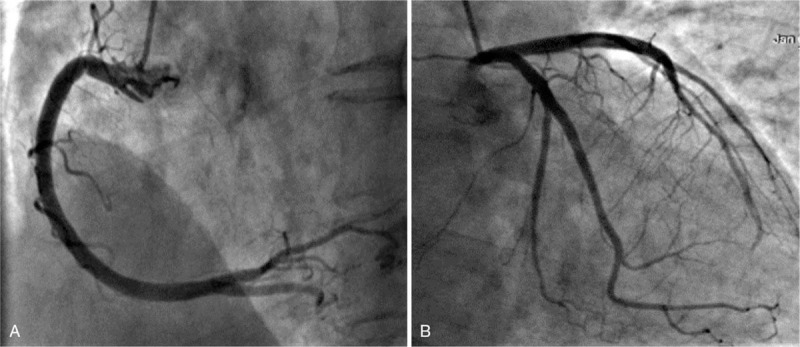
Coronary angiogram shows absence of obstruction in the coronary arteries.

Echocardiography showed left atrial distension, akinesis of the ventricular septum and left ventricular apical segments, hypokinesis of the left ventricular mid-segments, a left ventricular ejection fraction of 49%, and a slight elevation in pulmonary artery pressure of 30.2 mm Hg. Left ventriculogram during systole showed apical ballooning (Fig. 2) and ventricular wall motion abnormalities; cardiac catheterization did not reveal coronary atherosclerosis or significant lesions in the coronary arteries (Fig. 3). The diagnosis of TTS was retained, and supportive therapy was initiated. Two hours later, the patient's symptoms, which were similar to those of AMI, mitigated significantly and the ST segment returned to baseline. Cardiac troponin I was elevated (4.43 ng/mL; normal range <0.03) and decreased progressively thereafter (3.47 and 0.319 ng/mL; 3 and 48 hours later, respectively). Four days later, echocardiography showed normal systolic function without wall motion abnormalities and the patient returned to the orthopedics ward for further treatment.

## 3 Discussion

This case study highlights the recognition and risks of TTS during surgery under local anesthesia.

Although there is no consensus on the diagnostic criteria for TTS, the diagnostic criteria proposed by the Mayo Clinic have been widely accepted,^[1]^ which encompass the following features ^[4]^:

1. Transient hypokinesis, akinesis, or dyskinesis in the left ventricular mid-segments with or without apical involvement; abnormalities in regional wall motion that extend beyond single epicardial vascular distribution; a stressful trigger often (but not always) present.
2. Absence of obstructive coronary disease or angiographic evidence of acute plaque rupture.
3. New abnormalities on ECG (either ST segment elevation and/or T-wave inversion) or a modest elevation in cardiac troponin.
4. Absence of pheochromocytoma or myocarditis.

Patients with TTS show signs and symptoms that are similar to those of AMI, such as chest pain, dyspnea, ECG anomalies, and occasional syncope.^[2]^ In the present case, the primary presentation included chest pain, sweating, headache, and ST segment elevation on ECG, which was first considered to indicate possible AMI. Although the patient had a medical history of asthma, which can present with sweating owing to dyspnea, the other clinical manifestations outlined above are not typically associated with asthma. Dyskinesis and acute apical ballooning of the left ventricle and absence of acute plaque rupture support the diagnosis of TTS in our case.

The precise pathophysiology of TTS is not well known. Because of the correlation between stressful triggers and the onset of TTS, the adrenergic system is suspected to play a key role in its pathophysiology.^[5]^ In >85% of cases, either a physically or emotionally stressful event, which provokes the onset of TTS, can lead to the overproduction of endogenous catecholamines.^[6]^ A catecholamine surge is thought to contribute to apical ballooning through direct myocardial toxicity and/or disruption to the cardiac microvasculature.^[5]^ This surge is believed to promote vasospasm of both the epicardial vessels and the cardiac microvasculature, which contribute to an increased cardiac workload.^[7]^ Ultimately, the increased cardiac workload gives rise to a supply–demand mismatch and post-ischemic myocardial stunning.^[8]^ Furthermore, the administration of exogenous catecholamines can cause reversible TTS-like changes, which have been shown in animal models.^[9]^ The administration of high doses of catecholamines results in TTS in human patients.^[10]^ Serum catecholamine levels in patients with TTS are more than double those observed in patients with AMI, which demonstrates a correlation between TTS and increased catecholamine levels.^[11]^ This hyperadrenergic status is well-established in patients with a fracture who may experience acute pain when changing position. However, our case was observed during monitored anesthetic care (MAC) for thoracic vertebral fracture, which has rarely been reported. In our case, changing from a supine to a prone position may have caused acute severe pain owing to vertebral fracture, which could have aroused a catecholamine surge that ultimately induced a supply–demand mismatch and post-ischemic myocardial stunning.

TTS is unique cardiomyopathy that usually occurs in postmenopausal women. Approximately 90% of all reported cases have been in females,^[4]^ while the gender of the patient in the present case is male. Males are less likely to experience TTS than females, while males are more likely to die from TTS than females.^[12]^ Interestingly, patients with TTS precipitated by physical stressors have a higher likelihood of mortality than those precipitated by emotional stressors; and males are more likely to be precipitated by physical stressors.^[13]^ Actually, physical triggers and male sex are independent risk factors for mortality from TTS^[12]^ and clinicians should be aware of these independent risk factors.^[5]^

Clinically, TTS is an important disease that must be accurately distinguished from acute coronary syndrome to enable appropriate follow-up and medical management.^[14]^ Primary TTS was initially regarded as a benign condition with a good prognosis and complete recovery of ejection fraction in 95.7% of patients.^[15]^ However, in recent years, it has been realized that TTS can be associated with acute cardiac complications. These include cardiogenic shock, pulmonary edema, left ventricular outflow tract obstruction, and life-threatening arrhythmias, such as bradyarrhythmia, ventricular fibrillation, and sustained/non-sustained ventricular tachycardia.^[16,17]^ The in-hospital mortality from TTS was 4.5% (95% confidence interval [CI] 3.1–6.2)in a large meta-analysis of 2120 patients from 37 studies carried out in 11 different countries.^[17,18]^ Current treatment strategies for TTS mostly aim to reduce life-threatening complications, since no definitive benefit was yielded from pharmacological therapy, such as angiotensin-converting enzyme inhibitors, angiotensin II receptor blockers, and beta-blockers.^[14]^ This highlights the need for prospective randomized studies. A major goal in treating TTS is to reduce the patient's risk of major cerebrovascular events such as AMI and stroke, which occur in 7.1% of patients during the first 30 days of hospital admission.^[5,19]^ Anticoagulation should be used initially in patients with large areas of cardiac hypokinesis.^[20]^

In summary, it is necessary for anesthetists to recognize TTS which is life-threatening during MAC. We highlight the importance of being alerted to the possibility of TTS when managing patients with thoracic vertebral fractures undergoing surgery under local anesthesia. This case also informs anesthetists that they should consider the possibility of TTS when implementing field-block anesthesia, such as brachial, sciatic, or femoral plexus nerve block in patients with a fracture who may experience acute pain when changing position.

## Author contributions

**Conceptualization:** Zhen Zhang, Hao Kong.

**Data curation:** Zhen Zhang, Hao Kong.

**Formal analysis:** Zhen Zhang, Hao Kong, Ting-Ting Guan.

**Resources:** Zhen Zhang, Hao Kong, Si-Yu Zhang.

**Supervision:** Ting-Ting Guan.

**Writing – original draft:** Zhen Zhang, Hao Kong.

**Writing – review & editing:** Ting-Ting Guan.

## Abbreviations

Abbreviations: AMI = acute myocardial infarction, ECG = electrocardiography, MAC = monitored anesthetic care, PKP = percutaneous kyphoplasty, TTS = Takotsubo syndrome.
